# Residential household yard care practices along urban-exurban gradients in six climatically-diverse U.S. metropolitan areas

**DOI:** 10.1371/journal.pone.0222630

**Published:** 2019-11-13

**Authors:** Dexter H. Locke, Colin Polsky, J. Morgan Grove, Peter M. Groffman, Kristen C. Nelson, Kelli L. Larson, Jeannine Cavender-Bares, James B. Heffernan, Rinku Roy Chowdhury, Sarah E. Hobbie, Neil D. Bettez, Sharon J. Hall, Christopher Neill, Laura Ogden, Jarlath O’Neil-Dunne

**Affiliations:** 1 USDA Forest Service, Baltimore Field Station, Baltimore, MD, United States of America; 2 Florida Atlantic University, Center for Environmental Studies, Davie, FL, United States of America; 3 CUNY Advanced Science Research Center and Brooklyn College Department of Earth and Environmental Sciences 85 St. Nicholas Terrace, New York, NY, United States of America; 4 Department of Forest Resources and Department of Fisheries, Wildlife, & Conservation Biology, University of Minnesota, MN, United States of America; 5 Schools of Geographical Sciences and Urban Planning and Sustainability, Arizona State University, Tempe, AZ, United States of America; 6 Department of Ecology, Evolution and Behavior, University of Minnesota, St. Paul, MN, United States of America; 7 Nicholas School of the Environment, Duke University, Durham, NC, United States of America; 8 Graduate School of Geography, Worcester, MA, United States of America; 9 Cary Institute of Ecosystem Studies, Millbrook, NY, United States of America; 10 School of Life Sciences, Arizona State University, Tempe, AZ, United States of America; 11 The Woods Hole Research Center, Falmouth, MA, United States of America; 12 Dartmouth College, Department of Anthropology, Hanover, NH, United States of America; 13 University of Vermont, Spatial Analysis Lab, Rubenstein School of Environment and Natural Resources, Aiken Center, Burlington, VT, United States of America; Auburn University, UNITED STATES

## Abstract

Residential land is expanding in the United States, and lawn now covers more area than the country’s leading irrigated crop by area. Given that lawns are widespread across diverse climatic regions and there is rising concern about the environmental impacts associated with their management, there is a clear need to understand the geographic variation, drivers, and outcomes of common yard care practices. We hypothesized that 1) income, age, and the number of neighbors known by name will be positively associated with the odds of having irrigated, fertilized, or applied pesticides in the last year, 2) irrigation, fertilization, and pesticide application will vary quadratically with population density, with the highest odds in suburban areas, and 3) the odds of irrigating will vary by climate, but fertilization and pesticide application will not. We used multi-level models to systematically address nested spatial scales within and across six U.S. metropolitan areas—Boston, Baltimore, Miami, Minneapolis-St. Paul, Phoenix, and Los Angeles. We found significant variation in yard care practices at the household (the relationship with income was positive), urban-exurban gradient (the relationship with population density was an inverted U), and regional scales (city-to-city variation). A multi-level modeling framework was useful for discerning these scale-dependent outcomes because this approach controls for autocorrelation at multiple spatial scales. Our findings may guide policies or programs seeking to mitigate the potentially deleterious outcomes associated with water use and chemical application, by identifying the subpopulations most likely to irrigate, fertilize, and/or apply pesticides.

## Introduction

Residential areas are a dominant land use in urban regions, and yard care may produce substantial alterations in nutrient and hydrologic cycles, and general ecosystem structure and function [1]. These changes raise concern about the use of water, nitrogen, phosphorous [2, 3], and pesticide inputs, which may have unintended environmental consequences on beneficial insects and downstream water quality [4–6]. Lawns in the United States (U.S.) occupy 163,800 km^2^ (±35,850 km^2^), nearly four times as much land area as irrigated corn (43,000 km^2^), the country’s leading irrigated crop [7]. Although management actions may occur at the parcel or garden scale, the ecological outcomes may be expressed at larger scales [8]. Even small changes on individual properties across heterogeneous land managers and their practices [9, 10] could add up to continental-scale environmental changes. The apparent wide-spread nature of industrial lawncare, and the well-known associated negative environmental effects at the local-scale suggest a need to better understand the drivers, outcomes, and geographic variation in yard care practices, across the U.S.

Because irrigation, fertilization, and pesticide practices are not free, higher-income households may be more likely to irrigate, fertilize, and apply pesticides more than lower-income households [11–13]. Research in Baltimore, MD found positive correlations between household income and total yard care expenditures, expenditures on yard care supplies, and expenditure on yard machinery [14]. Yard care has also been found to vary by age, but the overall empirical evidence is mixed. In a study of Nashville, TN, Carrico et al. [15] found a modest, positive correlation between age and fertilizer use after controlling for property value, individualistic interests, environmental concerns and social pressures among other factors. In contrast, Martini and colleagues [16] did not find significant associations between age and fertilizer use or frequency of fertilizer application in a study of Minneapolis-St. Paul, MN when controlling for property size, home value, other sociodemographic variables, and cognitive and affective components. Therefore more research is needed to understand how age and yard care co-vary and by climate.

Households may also be consciously or subconsciously influenced by their neighbors [14, 15, 17–22]. One theory of residential behavior, the ecology of prestige [14, 17–19, 23], proposes that “household patterns of consumption and expenditure on environmentally relevant goods and services are motivated by group identity and perceptions of social status associated with different lifestyles” [14: 746]. Peer pressure and the household’s desire to fit in with their lifestyle group shape land management preferences [21]. Thus, different combinations of plantings and care practices reflect the different types of social groups and neighborhoods to which people belong [24, 25–27]. An indicator of the social pressures to uphold an established neighborhood aesthetic through yard care behaviors may be associated with whether a household knows their neighbors [27].

While yard care practices such as irrigation, fertilization and pesticide applications ultimately occur at the household-scale, household practices may also vary at other levels of aggregation including the neighborhood, municipal, regional, and climatic scales [28]. For instance, because residential land management may be affected by the space available for management [9, 19, 23, 29], population density may also be associated with yard care. This study therefore examines yard care in exurban, suburban, and urban Census block groups. The influence of precipitation, soil quality, and potential evapotranspiration affects the ability to grow grass, so this paper examines yard care in six regions with different soils and climates. We hypothesized that 1) income, age, and the number of neighbors known by name will be positively associated with the odds of having irrigated, fertilized, or applied pesticides in the last year, 2) irrigation, fertilization, and pesticide application will vary quadratically with population density, with the highest odds in suburban areas, and 3) the odds of irrigating will vary by climate (with irrigation more likely in hotter and drier regions), but fertilization and pesticide application will not vary by climate.

## Materials and methods

### Data and study areas

We investigated self-reported irrigation, fertilizer use and pesticide applications in six Metropolitan Statistical Areas (MSAs or regions) that cover major climatic regions of the US (Boston, MA; Baltimore, MD; Miami, FL; Minneapolis-St. Paul, MN; Phoenix, AZ; and Los Angeles, CA). Telephone interviews were stratified by population density (urban, suburban and exurban) as defined by the PRIZM geodemographic segmentation system [30]. PRIZM was chosen as a basis for sampling because prior research in Baltimore, MD [17] and New York, City [19] has shown that vegetation cover varies among the social groups PRIZM represents. We were therefore interested to see if yard management behaviors also varied by PRIZM segments [31]. Using these strata, >100,000 households were contacted between November 21 and December 29, 2011, across the six metropolitan regions, and ∼13,500 household were identified where the property contained a front or back yard and the respondent was over 18 years of age. From these households, approximately 70% of homeowners completed a 32 multi-part question telephone survey. Of the who people participated in the multi-part question telephone survey (n = 9,480), 7,317 respondents completed the questions that were the focus of this study, and indicated that they made their own decisions about yard management or about contracting yard care. Here we define yards as outdoor areas around homes inclusive of lawns and other types of groundcover and vegetation. Institutional Review Board approval was obtained from the University of Vermont Committee on Human Subjects (project CHRBS: B11-205). Also, we operationalize population density within Census block groups, and call MSAs regions. Our three dependent variables are irrigation, fertilizer use and pesticide application. Respondents were asked

“In the past year, which of the following has been applied to any part of your yard:

fertilizers?

pesticides to get rid of weeds or pests?

water for irrigating grass, plants or trees?”

The responses were coded as a binary 0 no, 1 yes (S1 Table). We specified a three-level binary logistic multi-level model for each of the three yard care behaviors. Respondents were also asked about their household income, which was recorded in 8 ordinal categories from 1 to 8 (<$15K, $15K - $25K, $25K - $35K, $35K - $50K, $50K - $75K, $75K - $100K, $100K - $150K, >$150K). The self-reported age of the respondent was recorded in five ordinal categories from 1 to 5 (<35 years old, 35 to 44, 45 to 54, 55 to 64, and > 65). Respondents were also asked “*About how many neighbors do you know by name*?” Answer choices included “*None*, *A few*, *About half*, *Most of them*, and *All of them*”, with responses also recorded as five ordinal categories from 1 to 5, respectively.

### Statistical analyses

In order to account for differences in the dependent variables by income, age and number of neighbors known by name, and simultaneously by population density and region, three-level binary logistic multi-level models (aka mixed models) were estimated for each dependent variable: irrigation, fertilization, and pesticide application. Multi-level modeling is a statistical technique designed to examine clustered, autocorrelated, or hierarchically nested datasets. Multi-level models accommodate observations from multiple scales and/or organizational levels simultaneously. Multi-level analyses are important because most related research with household-level research questions on yard care utilize neighborhood-level analyses, which are frequently operationalized with census block group data (e.g., [14, 17–19, 32–34], among others). In so doing, household-level heterogeneity may be averaged out, household-level questions may be mixed with block group-level analyses, and the research may be prone to the ecological fallacy [35]. Other studies focus on the household scale (e.g., [16, 20] among others), which precludes insights into multi-scaled relationships, and are susceptible to the atomistic fallacy. The atomistic fallacy occurs when individual samples are considered representative of aggregates of individuals, when they are not [36].

Consistent with best practices in multi-level modeling [37] we first specified a three-level null model for each of the three dependent variables. This approach first tests if a multi-level model is empirically warranted or not. The null model is:

$$\log\left(\frac{\pi_{ijk}}{1-\pi_{ijk}}\right)=\beta_{0jk}+v_{00k}+u_{0jk}$$ (Eq 1)

where π is the response from household *i* in Census block group *j* in metropolitan region *k*, and is the household-level intercept in level-two (block group) *j* in level-three (metropolitan region) *k*. Because the response variables are binary, the log odds of were modeled instead. No slopes are specified in Eq 1. *β*_0*jk*_ is decomposed and estimated as a hierarchically nested set of regression equations:

$$at\;Level\;2\;\left(Census\;block\;group\right):\;\beta_{0jk}=\delta_{00k}+u_{0jk}$$ (Eq 2)

$$at\;Level\;3\;\left(metropolitan\;region\right):\;\delta_{00k}=\gamma_{000}+v_{00k}$$ (Eq 3)

where *u*_0*jk*_ represents a neighborhood-scale random effect, and *v*_00*k*_ is a metropolitan regional-scale random effect. Level three consists of six units of analysis, whereas level 2 includes 18 units of analysis: six metropolitan regions times three levels of population density—urban, suburban, and exurban.

The null models demonstrated significant level 2 and 3 variation. Therefore, we next fit random intercept, fixed-slope models. This second set of models test the notion that the household lawn care behaviors vary systematically with neighborhood-level population density, and by MSA. After substituting Eqs 2 and 3 into Eq 1, and adding household-level independent variables, the random intercept, fixed-slope models estimated were:

$$\log\left(\frac{\pi_{ijk}}{1-\pi_{ijk}}\right)=\gamma_{000}+\sum_{s=3}\gamma_{s00}(x_{ijk}-\bar{x})+v_{00k}+u_{0jk}$$ (Eq 4)

Where *γ*_100_ is household income, *γ*_200_ is the respondent’s age, and *γ*_300_ the number of neighbors known by name. For all three practices the random intercept, fixed-slope models had better fit than their null model counterparts. For each of the three dependent variables (yard care behaviors), a more complex version of Eq 4 was also specified. These models add randomly varying slopes of the independent variables to Eq 4, at the neighborhood- and city-scales. However, the AIC scores were higher and therefore indicated worse model fit compared to Eq 4. Thus the models reported here reflect the specification in Eq 4; no added complexity is warranted for the random effects portion of the model beyond Eq 4., which contains random slopes at levels two and three (S2 Table). Once the random intercept, fixed-slope specification was chosen, a maximally interacting fixed effects portion was also fit for each dependent variable (S3 Table). In other words, the fixed effects portion of the model contained all possible interactions at the household level. None of the interactions were significant, and the output is omitted for brevity (S4 Table). One exception is for the Income by Age interaction in the Pesticide Application model which yielded a modest (OR = 0.97, 95% CI [0.95 to 0.99]) relationship.

In summary, nine models were fit (three dependent variables, times three specifications: the null model shown in Eq 1, a random intercept, fixed-slope model shown in Eq 4, and random intercept, random slope model not shown for brevity, (S2 Table). All statistical analyses were conducted using the free R programing language version 3.0.2 [38]. Multi-level models were fit using the lme4 package version 1.1–4 [39, 40], and significance tests were carried out with the lmerTest package version 2.0–6 [41].

## Results

The average reported income (5.6) and median income (6.0) score corresponds to the $75K - $100K group (Table 1). Similarly for age, the mean (3.34) and median (3.0) correspond to the 45 to 54 age group. Finally, the average (2.96) of number of neighbors known by name corresponds to the *“About half”* answer, although the median (3) corresponds to the *“Most of them”* answer choice.

**Table 1:** Descriptive statistics of the independent variables: Income, age, and number of neighbors known by name in six metropolitan areas of the United States.

Thematic group	Variable	Mean (%)	sd	Min.	Max.	Spearman’s ρ Correlations	
						**2**	**3**
Socio-economic Status	**1** Income ^a^	5.59	1.75	1	8	-0.2***	0.15***
Lifestage	**2** Age of respondent ^b^	3.34	1.22	1	5	1	0.02
Neighborhood cohesion	**3** # of neighbors known by name ^b^	2.96	1.01	1	5		1

*** p < 0.001, ** p < 0.01, * p < 0.05

^a^ eight ordinal categories

^b^ five ordinal categories

n = 7,317

### Irrigation

Irrigation was the most common yard care practice in our study, 80% of residents reported having irrigated their yard in the last year. Irrigation was positively and significantly correlated with income (ρ = 0.11, p < 0.001) and the number of neighbors known by name (ρ = 0.03, p < 0.01), but not age (ρ = 0.00, p = 0.90; Table 1). The range in irrigation by metropolitan region varied from 64% in Baltimore to 92% in Los Angeles (Table 2). When parsed by the three population density categories (urban, suburban, and exurban) per metropolitan region, exurban respondents in Baltimore had the lowest proportion of irrigation (60%), and exurban communities in Phoenix had the greatest proportion of irrigators (94%, Table 2).

**Table 2:** Self-reported irrigation, fertilization, and pesticide application proportions by population density and metropolitan region.

Region	n	Population Density	n	% Irrigation (Region)	% Irrigation (Population Density)	% Fertilization (Region)	% Fertilization (Population Density)	% Pesticide Application (Region)	% Pesticide Application (Population Density)
Phoenix (CAP)	1,289	Urban	302	90	84	60	57	66	52
		Suburban	592		90		64		68
		Exurban	395		94		57		73
Los Angeles	1,128	Urban	246	92	87	70	55	45	39
		Suburban	629		93		74		46
		Exurban	253		92		74		47
Minne-St. Paul (CDR)	1,319	Urban	311	85	84	70	45	53	33
		Suburban	584		87		80		58
		Exurban	424		83		74		61
Baltimore (BES)	1,240	Urban	266	64	66	54	43	48	39
		Suburban	574		65		62		51
		Exurban	400		60		49		48
Boston (PIE)	1,247	Urban	242	71	72	63	48	42	29
		Suburban	621		71		65		44
		Exurban	384		71		70		48
Miami (FCE)	1,094	Urban	296	78	71	67	56	63	51
		Suburban	523		84		72		65
		Exurban	275		73		68		72

The regression-adjusted estimates reinforce the descriptive and bivariate associations reported just above. For example, household income and the number of neighbors known by name (reflecting cohesion) were positively and significantly associated with irrigation when controlling for metropolitan region and block group-level population density (Table 3), while respondent age did not exhibit a significant relationship with irrigation. Specifically, households with higher incomes were on average 1.23 times more likely (95% CI [1.19, 1.27]) to report that they irrigated their yards in the last year. Note that 1.23 is an odds ratio (OR), which is simply the probability of an event divided by one minus that same probability. Therefore probabilities and ORs measure the same phenomenon on different scales. Odds ratios are easy to interpret; an OR of 1.23 corresponds to an increase of 23% in the odds of irrigating with a one-unit increase in income above the average. The odds of irrigating were 8% larger (95% CI [1.02, 1.16]) when homeowners knew more neighbors by name.

**Table 3:** Three-level binary logistic regression outputs for irrigation, fertilization, and pesticide application.

	Irrigation			Fertilization			Pesticide Application		
* *	*Odds Ratio*	*95% CI*	*p*	*Odds Ratio*	*95% CI*	*p*	*Odds Ratio*	*95% CI*	*p*
**Fixed Parts**									
Intercept (*γ*_000_)	4.64	2.64 to 8.14	**< .001**	1.72	1.36 to 2.18	**< .001**	1.07	0.75 to 1.52	.706
Income (*γ*_100_)	1.23	1.19 to 1.27	**< .001**	1.22	1.19 to 1.26	**< .001**	1.16	1.12 to 1.19	**< .001**
Age (*γ*_200_)	1.03	0.98 to 1.09	.213	1.09	1.05 to 1.14	**< .001**	0.99	0.95 to 1.03	.715
# of neighbors known by name (*γ*_300_)	1.09	1.02 to 1.16	**.007**	1.09	1.04 to 1.15	**< .001**	1.00	0.95 to 1.05	.984
**Random Parts**									
τ_00, CityPD_	0.064			0.187			0.129		
τ_00, CityLab_	0.466			0.021			0.146		
N_CityPD_	18			18			18		
N_CityLab_	6			6			6		
ICC_CityPD_	0.017			0.053			0.036		
ICC_CityLab_	0.122			0.006			0.041		
Observations	7317			7317			7317		
Deviance	6,700.347			9,026.196			9,610.827		

Bold indicates significant at 95% level

**Fig 1:**
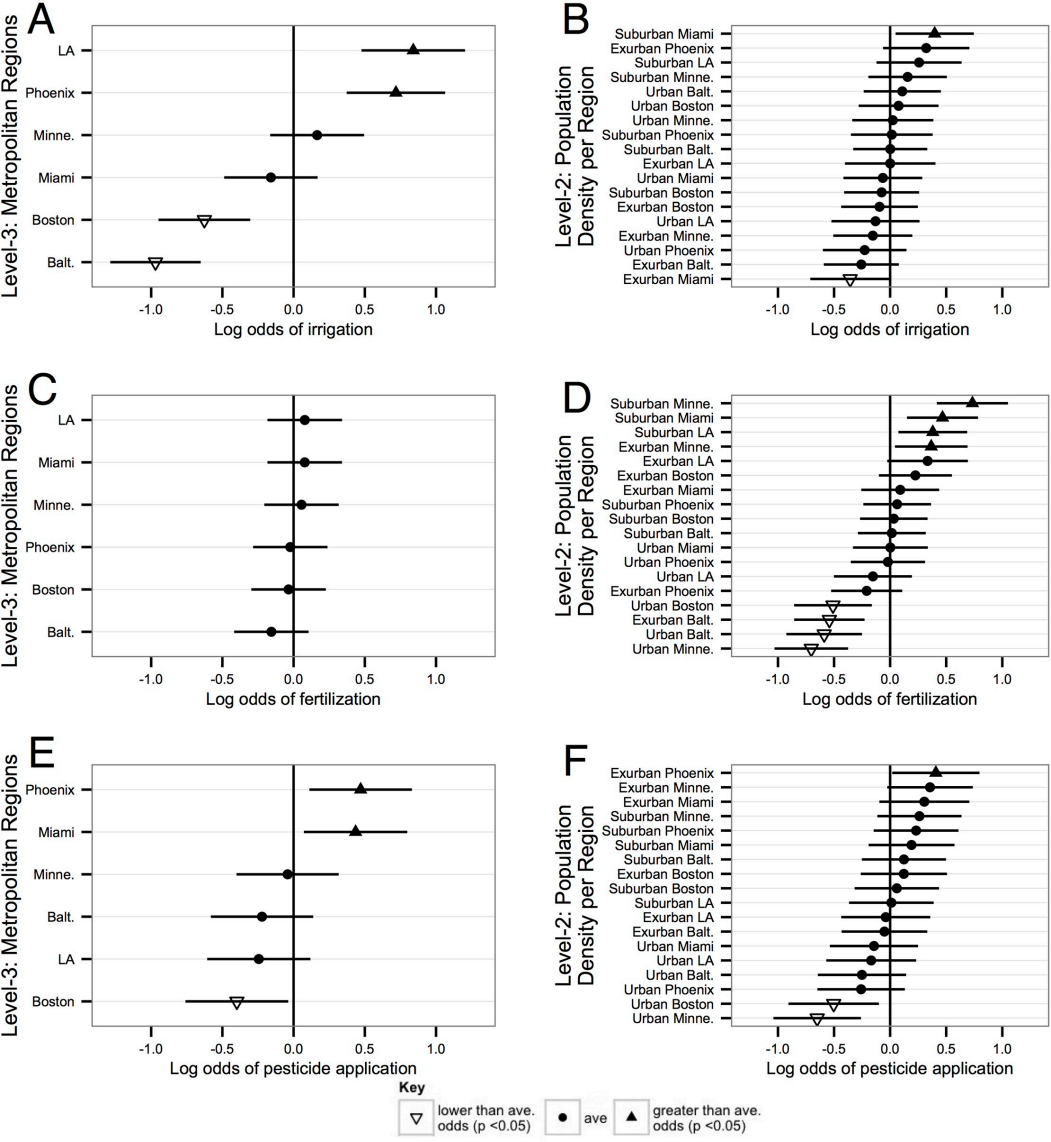
Dots and triangles represent the point estimates for the random effects for block group and metropolitan regions, *v*_00*k*_ and *u*_0*jk*_, respectively. Horizontal lines are the 95% confidence intervals. When the interval crosses the mean (zero) the estimate is not significantly different from the mean, which is shown with dots. When there are significant differences below the mean, they are shown with downward-oriented triangles, while positive differences above the mean are shown with upward-facing triangles. Regions and region-population density combinations are arrayed according to their log odds (lowest on bottom, highest on top); note differences in y-axes across panels.

The odds of having irrigated in the last year vary across the examined metropolitan regions in distinct climate zones (Fig 1A). As expected, households in the hot, dry climates (Los Angeles and Phoenix) were significantly more likely to water their yards than their counterparts in the cooler, wetter metropolitan areas of Boston and Baltimore. The odds of irrigating in Minneapolis-St. Paul and Miami reflect the sample average, after controlling for population density and the household-level predictors. In contrast, household-level relationships were fairly homogenous at the block group-scale, after the effects of the metropolitan regions were incorporated (Fig 1B). Of the 18 metropolitan region-population density combinations, just two had intercepts that varied from the average joint effect for the full sample. Specifically, households in suburban Miami were more likely to irrigate their yards than the other population density categories, and households in exurban Miami were significantly less likely to water (Fig 1B). The remaining 16 combinations showed no variation at that scale.

### Fertilization

Fertilization was less common than irrigation in our sample, with 64% of all respondents having applied fertilizer in the past year (Table 2). Fertilization was positively and significantly correlated with income (ρ = 0.15, p < 0.001), age (ρ = 0.04, p < 0.001), and the number of neighbors known by name (ρ = 0.07, p < 0.001; Table 1). The relative proportion of households engaging in these yard care practices varied by metropolitan region, with Baltimoreans exhibiting the lowest proportion of households applying fertilizers (54%) and Minneapolitans and Angelinos the greatest (70%, Table 2). The application of fertilizers was least prevalent among households in urban Baltimore (43%), and most prevalent in suburban Minneapolis-St. Paul (80%, Table 2).

All three household-level independent variables exhibited statistically significant, positive relationships with fertilization. The odds ratio for income and fertilization (1.22, 95% CI [1.19, 1.26]) is very similar to the previously noted positive relationship between income and irrigation. Age and number of known neighbors increased the odds of fertilization, with odds ratios of 1.09, 95% CIs [1.05, 1.14] and [1.04, 1.15], respectively.

The odds of fertilization in suburban Minneapolis-St. Paul, Miami, Los Angeles and in exurban Minneapolis-St. Paul are significantly higher than in urban Boston, Minneapolis, Baltimore, and exurban Baltimore. The remaining ten metropolitan regional-population density combinations exhibit similar, and average, probabilities of applying fertilizers (Fig 1D). Greater, and statistically significant, variation is observed across an urban-exurban gradient at the block group-scale, than at the regional-scale. There was no variation among metropolitan regions, indicating relative homogeneity at that scale for applying fertilizers after accounting for population density and household-level predictors (Fig 1C).

### Pesticide application

Pesticide application was the least prevalent yard care activity reported in our responses: 53% of all respondents indicated that they applied pesticides to eliminate weeds or pests in the last year. Income was an important correlate of this practice, but it was also the only household-level variable that was statistically significantly related to pesticide use, with wealthier respondents more likely to apply pesticides (OR = 1.16, 95% CI [1.12, 1.19]). Relatively little variation was observed across block group-scale population density classes.

Similar to irrigation and fertilization yard care practices, the prevalence of household pesticide applications by block group-scale population density classes revealed geographic differences. However, those differences were evident in only 3 of the 18 cases: urban Minneapolis-St. Paul and Boston exhibited a significantly lower proportion of households that applied pesticides, while households in the exurban Phoenix region were more likely to apply pesticides (Fig 1F). Regional-scale results show that more residents in Phoenix and Miami, and fewer residents in Boston, applied pesticides, compared to the other regions, when controlling for the household-level predictors (Fig 1E and 1F).

## Discussion

Because yard care practices examined here have financial costs, we expected that higher income-earning households would be more likely to irrigate and apply fertilizers and pesticides than lower-income households. We found that higher-income households were more likely to report irrigation, fertilization and pesticide application than lower-income households (~16% to 23% greater odds, Table 3), after adjusting for age, known neighbors, population density and regional influences. Income was the only household-level variable that was statistically significant across all models for yard care practices, so yard care practices may be cost-prohibitive for some households who wish to obtain a well-manicured aesthetic.

Yard care behaviors have also been hypothesized to vary with the resident’s age. Previous research on the relationship between age and yard care practices revealed mixed findings. Some researchers have suggested the capacity for yard care decreases with increasing age [24, 42, 43], while other studies employing multivariate analyses have revealed no significant relationships [i.e. 15, 16]. In this systematic, multi-site comparative sample, we found a ~9% increase in the odds of fertilizing with increased age, but no significant associations between age and irrigation or pesticide application.

The relationship between age and yard care could be positive for some age classes or lifestages and negative for others, which would explain a null finding. Time and money might be limiting factors for younger households. As a household ages there could be more available time and potentially more money, while retirement may lead to even more available time but fewer financial resources to invest in yard care. It is also possible that older and higher-income households are more likely to hire yard care service companies to perform these tasks, which would diminish the argument that ability declines with age. A study that did not find age to have a significant relationship with fertilizing frequency also found a significant and positive association with lawn care services [16]. Specifically, it cannot be assumed that the homeowner does the yard work. Thus, age may not be a predictor of capacity. Since our survey did not specifically identify who does the work of yard care, we are unable to further disentangle this relationship. The influence of age needs to be better understood in research theorizing and empirically documenting urban residential ecologies.

In addition to income and age, peer pressure may also influence yard care practices. Given the abundant literature on social norms and landscaping [14, 15, 17–22], we hypothesized a significant and positive relationship between the number of neighbors known by name and the use of water, fertilizer, and pesticides. We found that knowing more neighbors by name corresponded to an ~9% increase in the odds of both irrigation and fertilization, but no significant difference in the odds of applying pesticides. This result may suggest that the desire to fit in and conform to neighborhood norms may increase when a household knows its neighbors by maintaining a neighborhood aesthetic through certain, but not all, yard care behaviors.

The relationships between income, age, the number of neighbors known by name varied across and urban to exurban gradients, and among regions in different climates. Our multi-scale analyses indicated that the odds of irrigation were statistically indistinguishable across population density categories, but variable across regions. Similarly, our multi-level analyses were important for uncovering the variability in odds of fertilizer practices across an urban-exurban gradient. Our multi-level, multivariate analyses indicate which specific management practices are associated with which scales, and how they are linked to particular sets of behavioral drivers.

### Limitations

This research was limited by the coarse yes/no telephone survey questions for yard care practices. In-depth surveys and interviews are likely better for obtaining information about the intensity of yard care practices and contextual factors affecting them (e.g. [16, 20, 44, 45]). Subsequent research could examine the age of who actually carries out these practices, possible interaction effects with income, as well as the frequency, amount, location (i.e., part or whole yard), and timing (i.e., seasonality) of yard care practices. Collecting this type of information would also enable a more explicit connection to household-level environmental outcomes rather than only household-level behaviors. Yard care behaviors studied here are well-correlated with each other (Table 1). A next step could be to examine a multi-variate, multi-level, multi-site model akin to a multiple analysis of variance with household-level fixed effects. We specified simpler models for ease of interpretation, to build on the bivariate approach adopted elsewhere [8], and to establish the utility of the multi-level analytical strategy.

### Management implications

We examined patterns in yard care practices to advance understanding of residential ecosystems, because these landscapes are increasing in area and have complex environmental impacts. We used a multi-management practice, multi-site, and multi-level approach in this paper to investigate how income, age, and the number of neighbors known by name relate to three common yard management practices: irrigation, fertilization, and pesticide application, across household, block group, and regional scales. We found that knowing more neighbors by name is associated with elevated odds of irrigating and fertilizing. Neighborhood social networks may therefore be important for planners and government agencies seeking to influence residents to use more environmentally-friendly alternatives to traditional landscaping [46, 47]. Moreover, fertilization is more likely among older residents. Managers seeking to reduce fertilizer use may therefore focus on older social groups for more effective targeting, whether they hire lawn care companies or do the yard work themselves. Previous efforts in Wisconsin have shown the effectiveness of policies that reduce phosphorus pollution as much as 37% from agricultural and urban runoff from 1995 to 2007 [48]. Future research should continue to investigate the geographic variation, drivers, and outcomes of yard care.

## Supporting information

S1 Table Survey questions and their answer choices. (DOCX)

S2 Table Multi-level regression models. (DOCX)

S3 Table Three-level binary logistic regression outputs for irrigation, fertilization, and pesticide application with maximal fixed effects. (HTML)

S4 Table Three-level binary logistic regression outputs for respondents who irrigate, fertilize, and apply pesticides. (HTML)
