# Supplementary material for: Residential household yard care practices along urban-exurban gradients in six climatically-diverse U.S. metropolitan areas
Source: PLoS One. 2019 Nov 13;14(11):e0222630. doi: 10.1371/journal.pone.0222630 (PMC6853287; doi:10.1371/journal.pone.0222630)
Supplement: S1 Table — (DOCX) [file pone.0222630.s001.docx]

S1 Table. Survey questions and their answer choices.

| **Domain** | **Question** | **Answer Choices** |
| --- | --- | --- |
| Fertilizer use | In the past year, which of the following has been applied to any part of your yard: | fertilizers? (1 = yes, 0 = no) |
| Pesticide application |  | pesticides to get rid of weeds or pests? (1 = yes, 0 = no) |
| Irrigation use |  | water for irrigating grass, plants or trees? (1 = yes, 0 = no) |
| Income | And lastly, is the total annual income of all members of your household over $50,000 or under $50,000? | -2 Over $50,000  -3 Under $50,000  RF/DK  b. If over $50K, is it:  1 $50,000 - $75,000  2 $75,000 - $100,000  3 $100,000 - $150,000  4 OVER $150,000?  c: If under $50K, is it:  4 $35,000 - $50,000,  3 between $25,000 - $35,000,  2 $15,000 to $25,000, or  1 under $15,000? |
| Age | Please stop me when I reach the category that includes your age. Are you: | -1 under 35,  -2 35 to 44,  -3 45 to 54,  -4 55 to 64, or  -5 65 or over?  RF (recoded as NA) |
| Known neighbors | A bout how many neighbors do you know by name? | -1 none,  -2 a few,  -3 about half,  -4 most of them, or  -5 all of them?  DK/RF (recoded as NA) |
