# Supplementary material for: Residential household yard care practices along urban-exurban gradients in six climatically-diverse U.S. metropolitan areas
Source: PLoS One. 2019 Nov 13;14(11):e0222630. doi: 10.1371/journal.pone.0222630 (PMC6853287; doi:10.1371/journal.pone.0222630)
Supplement: S2 Table — (DOCX) [file pone.0222630.s002.docx]

S2 Table. Multi-level regression models.

| Model name | Specification | R code | Greek |
| --- | --- | --- | --- |
| Null model | unconditional | glmer(q6_3 ~ 1 + (1\| CityPD) + (1 \| CityLab), data = tbl, family=binomial(link = "logit")) | $\log\left( \frac{\boldsymbol{\pi}_{\boldsymbol{ijk}}}{\boldsymbol{1-}\boldsymbol{\pi}_{\boldsymbol{ijk}}} \right)\boldsymbol{=}\boldsymbol{\beta}_{\boldsymbol{0}\boldsymbol{jk}}\boldsymbol{+}\boldsymbol{v}_{\boldsymbol{00}\boldsymbol{k}}\boldsymbol{+}\boldsymbol{u}_{\boldsymbol{0}\boldsymbol{jk}}$ |
| Model 1 | random intercept, fixed slope | glmer(q6_3 ~ scale(INCOME, center=T, scale=F) + (q26_1, center=T, scale=F) + scale(q16_1, center=T, scale=F) + (1 \| CityPD) + (1 \| CityLab), data = tbl, family=binomial(link = "logit")) | $\log\left( \frac{\boldsymbol{\pi}_{\boldsymbol{ijk}}}{\boldsymbol{1-}\boldsymbol{\pi}_{\boldsymbol{ijk}}} \right)\boldsymbol{=}\text{γ}_{\boldsymbol{000}}\boldsymbol{+}\sum_{\boldsymbol{s=3}} \text{γ}_{\boldsymbol{s}\boldsymbol{00}}\left( \boldsymbol{x}_{\boldsymbol{ijk}}\boldsymbol{-}\bar{\boldsymbol{x}} \right)\boldsymbol{+}\boldsymbol{v}_{\boldsymbol{00}\boldsymbol{k}}\boldsymbol{+}\boldsymbol{u}_{\boldsymbol{0}\boldsymbol{jk}}$ |
| Model 2 | fixed intercept, random slope | glmer(q6_3 ~ 1 + (scale(INCOME, center=T, scale=F) + scale(q26_1, center=T, scale=F) +scale(q16_1, center=T, scale=F \| CityPD) + (1 \| CityLab), data = tbl, family=binomial(link = "logit")) | $\log\left( \frac{\boldsymbol{\pi}_{\boldsymbol{ijk}}}{\boldsymbol{1-}\boldsymbol{\pi}_{\boldsymbol{ijk}}} \right)\boldsymbol{=}\text{γ}_{\boldsymbol{000}}\boldsymbol{+}\sum_{\boldsymbol{s=3}} \boldsymbol{v}_{\boldsymbol{s}\boldsymbol{0}\boldsymbol{k}}\left( \boldsymbol{x}_{\boldsymbol{ijk}}\boldsymbol{-}\bar{\boldsymbol{x}} \right)\boldsymbol{+}\boldsymbol{u}_{\boldsymbol{0}\boldsymbol{jk}}$ |
| Model 3 | Random intercept, random slope | glmer(q6_3 ~ scale(INCOME, center=T, scale=F) + scale(q26_1, center=T, scale=F) + scale(q16_1, center=T, scale=F) + (scale(INCOME, center=T, scale=F) + scale(q26_1, center=T, scale=F) + scale(q16_1, center=T, scale=F) \| CityPD) + (1 \| CityLab), data = tbl, family=binomial(link = "logit")) | $\log\left( \frac{\boldsymbol{\pi}_{\boldsymbol{ijk}}}{\boldsymbol{1-}\boldsymbol{\pi}_{\boldsymbol{ijk}}} \right)\boldsymbol{=}\text{γ}_{\boldsymbol{000}}\boldsymbol{+}\sum_{\boldsymbol{s=3}} \text{γ}_{\boldsymbol{s}\boldsymbol{00}}\left( \boldsymbol{x}_{\boldsymbol{ijk}}\boldsymbol{-}\bar{\boldsymbol{x}} \right)\boldsymbol{+}\sum_{\boldsymbol{s=3}} \boldsymbol{v}_{\boldsymbol{s}\boldsymbol{0}\boldsymbol{k}}\left( \boldsymbol{x}_{\boldsymbol{ijk}}\boldsymbol{-}\bar{\boldsymbol{x}} \right)\boldsymbol{+}\boldsymbol{u}_{\boldsymbol{0}\boldsymbol{jk}}$ |
