# Supplementary material for: Residential household yard care practices along urban-exurban gradients in six climatically-diverse U.S. metropolitan areas
Source: PLoS One. 2019 Nov 13;14(11):e0222630. doi: 10.1371/journal.pone.0222630 (PMC6853287; doi:10.1371/journal.pone.0222630)
Supplement: S3 Table — (HTML) [file pone.0222630.s003.html]

|  |  |  |  |  |  |  |  |  |  |  |  |  |  |  |  |  |  |  |  |  |
| --- | --- | --- | --- | --- | --- | --- | --- | --- | --- | --- | --- | --- | --- | --- | --- | --- | --- | --- | --- | --- |
|  |  | Irrigation | | |  | Irrigation | | |  | Fertilization | | |  | Fertilization | | |  | Pesticide Application | | |
|  |  | Odds Ratio | 95% CI | p |  | Odds Ratio | 95% CI | p |  | Odds Ratio | 95% CI | p |  | Odds Ratio | 95% CI | p |  | Odds Ratio | 95% CI | p |
| Fixed Parts | | | | | | | | | | | | | | | | | | | | |
| (Intercept) |  | 4.64 | 2.64 to 8.14 | **<.001** |  | 4.64 | 2.63 to 8.17 | **<.001** |  | 1.72 | 1.36 to 2.18 | **<.001** |  | 1.71 | 1.35 to 2.17 | **<.001** |  | 1.06 | 0.75 to 1.51 | .746 |
| Income |  | 1.23 | 1.19 to 1.27 | **<.001** |  | 1.23 | 1.19 to 1.27 | **<.001** |  | 1.22 | 1.19 to 1.26 | **<.001** |  | 1.23 | 1.19 to 1.26 | **<.001** |  | 1.16 | 1.13 to 1.20 | **<.001** |
| Age |  | 1.03 | 0.98 to 1.09 | .213 |  | 1.02 | 0.97 to 1.08 | .416 |  | 1.09 | 1.05 to 1.14 | **<.001** |  | 1.09 | 1.04 to 1.13 | **<.001** |  | 0.99 | 0.95 to 1.03 | .614 |
| # of neighbors known by name |  | 1.09 | 1.02 to 1.16 | **.007** |  | 1.08 | 1.02 to 1.15 | **.015** |  | 1.09 | 1.04 to 1.15 | **<.001** |  | 1.09 | 1.03 to 1.15 | **.001** |  | 0.99 | 0.95 to 1.04 | .786 |
| Income x Age |  |  |  |  |  | 0.98 | 0.96 to 1.01 | .232 |  |  |  |  |  | 0.98 | 0.96 to 1.01 | .201 |  | 0.97 | 0.95 to 0.99 | **.016** |
| Income x # of Known Neighbors |  |  |  |  |  | 0.99 | 0.95 to 1.02 | .390 |  |  |  |  |  | 1.01 | 0.98 to 1.04 | .511 |  | 1.00 | 0.97 to 1.03 | .991 |
| Age x # of Known Neighbors |  |  |  |  |  | 0.96 | 0.92 to 1.02 | .168 |  |  |  |  |  | 0.98 | 0.94 to 1.02 | .273 |  | 0.98 | 0.94 to 1.02 | .353 |
| Income x Age x # of Known Neighbors |  |  |  |  |  | 0.99 | 0.97 to 1.02 | .620 |  |  |  |  |  | 0.99 | 0.97 to 1.01 | .404 |  | 0.98 | 0.96 to 1.01 | .163 |
| Random Parts | | | | | | | | | | | | | | | | | | | | |
| τ00, CityPD |  | 0.064 | | |  | 0.063 | | |  | 0.187 | | |  | 0.184 | | |  | 0.125 | | |
| τ00, CityLab |  | 0.466 | | |  | 0.472 | | |  | 0.021 | | |  | 0.022 | | |  | 0.147 | | |
| NCityPD |  | 18 | | |  | 18 | | |  | 18 | | |  | 18 | | |  | 18 | | |
| NCityLab |  | 6 | | |  | 6 | | |  | 6 | | |  | 6 | | |  | 6 | | |
| ICCCityPD |  | 0.017 | | |  | 0.016 | | |  | 0.053 | | |  | 0.053 | | |  | 0.035 | | |
| ICCCityLab |  | 0.122 | | |  | 0.123 | | |  | 0.006 | | |  | 0.006 | | |  | 0.041 | | |
| Observations |  | 7317 | | |  | 7317 | | |  | 7317 | | |  | 7317 | | |  | 7317 | | |
| AIC |  | 6771.618 | | |  | 6775.413 | | |  | 9107.270 | | |  | 9111.268 | | |  | 9693.146 | | |
| Deviance |  | 6700.347 | | |  | 6696.352 | | |  | 9026.196 | | |  | 9022.367 | | |  | 9602.818 | | |
