# Supplementary material for: Residential household yard care practices along urban-exurban gradients in six climatically-diverse U.S. metropolitan areas
Source: PLoS One. 2019 Nov 13;14(11):e0222630. doi: 10.1371/journal.pone.0222630 (PMC6853287; doi:10.1371/journal.pone.0222630)
Supplement: S4 Table — (HTML) [file pone.0222630.s004.html]

|  |  |  |  |  |  |  |  |  |
| --- | --- | --- | --- | --- | --- | --- | --- | --- |
|  |  | supper user: does all 3 | | |  | supper user: does all 3 | | |
|  |  | Odds Ratio | 95% CI | p |  | Odds Ratio | 95% CI | p |
| Fixed Parts | | | | | | | | |
| (Intercept) |  | 0.53 | 0.38 to 0.74 | **<.001** |  | 0.53 | 0.38 to 0.74 | **<.001** |
| Income |  | 1.23 | 1.19 to 1.26 | **<.001** |  | 1.23 | 1.19 to 1.27 | **<.001** |
| Age |  | 1.00 | 0.95 to 1.04 | .835 |  | 0.99 | 0.95 to 1.04 | .804 |
| # of neighbors known by name |  | 1.07 | 1.02 to 1.12 | **.009** |  | 1.07 | 1.01 to 1.12 | **.015** |
| Income x Age |  |  |  |  |  | 0.98 | 0.96 to 1.01 | .127 |
| Income x # of Known Neighbors |  |  |  |  |  | 0.99 | 0.97 to 1.02 | .719 |
| Age x # of Known Neighbors |  |  |  |  |  | 0.97 | 0.93 to 1.01 | .154 |
| Income x Age x # of Known Neighbors |  |  |  |  |  | 0.99 | 0.97 to 1.02 | .492 |
| Random Parts | | | | | | | | |
| τ00, CityPD |  | 0.136 | | |  | 0.132 | | |
| τ00, CityLab |  | 0.124 | | |  | 0.125 | | |
| NCityPD |  | 18 | | |  | 18 | | |
| NCityLab |  | 6 | | |  | 6 | | |
| ICCCityPD |  | 0.038 | | |  | 0.037 | | |
| ICCCityLab |  | 0.035 | | |  | 0.035 | | |
| Observations |  | 7317 | | |  | 7317 | | |
| AIC |  | 9207.329 | | |  | 9210.027 | | |
| Deviance |  | 9126.287 | | |  | 9121.363 | | |
